# Unraveling the Heterogeneity of Deficiency of Mismatch Repair Proteins in Endometrial Cancer: Predictive Biomarkers and Assessment Challenges

**DOI:** 10.3390/cancers16203452

**Published:** 2024-10-11

**Authors:** Filomena M. Carvalho, Jesus P. Carvalho

**Affiliations:** 1 Department of Pathology, Faculdade de Medicina da Universidade de Sao Paulo, São Paulo 01246-903, Brazil; 2 Department of Obstetrics and Gynecology, Instituto do Cancer do Estado de Sao Paulo, Faculdade de Medicina da Universidade de Sao Paulo, São Paulo 01246-903, Brazil; jpcarvalho@usp.br

**Keywords:** endometrial cancer, biomarkers, molecular classification, mismatch repair proteins, prognosis, predictive factors

## Abstract

**Simple Summary:**

Endometrial cancer is becoming more common globally, mainly due to factors such as aging populations and obesity. Despite the early detection in most cases, a significant number of patients still face advanced stages of the disease, leading to higher mortality rates compared to previous years. Recent advances in treatment, such as immunotherapy, are offering new hope, especially for patients with specific genetic abnormalities in their tumors causing microsatellite instability. However, not all patients with these genetic markers, called mismatch repair protein deficiency, respond equally well to immunotherapy. This fact highlights the need for better ways to predict who will benefit the most from these treatments. This review focuses on understanding the biology of mismatch repair proteins, their variability, the challenges in testing them, and their potential in predicting treatment outcomes in endometrial cancer.

**Abstract:**

Endometrial cancer (EC) poses a significant global health challenge, with increasing prevalence in 26 of 43 countries and over 13,000 deaths projected in the United States by 2024. This rise correlates with aging populations, the obesity epidemic, and changing reproductive patterns, including delayed childbearing. Despite the early diagnosis in 67% of cases, approximately 30% of cases present with regional or distant spread, leading to nearly 20% mortality rates. Unlike many cancers, EC mortality rates are escalating, outpacing therapeutic advancements until recently. One of the reasons for this was the lack of effective therapeutic options for advanced disease until recently. The introduction of immunotherapy has marked a turning point in EC treatment, particularly benefiting patients with defects in mismatch repair proteins (dMMRs). However, dMMR status alone does not ensure a favorable response, underscoring the need for precise patient selection. This review explores the pivotal role of mismatch repair proteins in EC, emphasizing their heterogeneity, the challenges in their assessment, and their potential as predictive biomarkers.

## 1. Introduction

Uterine corpus cancer, represented mainly by endometrial carcinoma (EC), accounts for 7% of female cancers in the United States, with 66,200 new cases in 2023 [1]. While cervical cancer incidence is declining, the prevalence of corpus cancer is rising worldwide due to aging populations, the obesity epidemic, and a decline in fertility [1]. The International Agency for Research on Cancer (IARC) estimates 420,242 new cases and 97,704 deaths in 2022 across the world [2]. Although 67% of cases present as a localized disease at diagnosis, the mortality rate is increasing, with 13,030 deaths estimated in 2023 in the United States, representing 5% of all female deaths for cancer [1,3]. These numbers are similar to those of ovarian cancer, previously considered the most aggressive female cancer, but for patients with ovarian cancer, there is an improvement in therapeutic options. In our experience, which is not different from the literature, EC presented in FIGO 2009 stages III and IV in 37.6% of cases, 17.7% with positive lymph nodes, and 17% of patients died from the disease [4]. Until recently, the better option we had for first-line systemic therapy for recurrent/advanced disease was carboplatin/paclitaxel based on the phase III trial NRG Oncology/GOG0209, which demonstrated the noninferiority of this regimen compared to the classical paclitaxel–doxorubicin–cisplatin regime. The benefit of this regimen, although it showed lower toxicity, was far from ideal, with about 65% of deaths within a median follow-up of 124 months [5]. After the molecular insights obtained from the Cancer Genome Atlas (TCGA) and the identification of a substantial group with tumors with microsatellite instability, immunotherapy was introduced for the treatment of EC, and we can say that this was the turning point for the improvement in the treatment outcomes of recurrent and advanced disease [6,7].

In this review, we present the molecular classification that has improved our understanding of the different subtypes of EC, and we explore the many facets of the microsatellite instability (MSI) subgroup. We briefly present the studies that support the indication of checkpoint inhibitors (ICIs) in the treatment of recurrent/advanced disease of EC and the pivotal role of mismatch repair (MMR) proteins leading to MSI, emphasizing their heterogeneity, the challenges in their assessment, and their potential as predictive biomarkers. Understanding the heterogeneity within this subgroup is crucial for improving diagnostic accuracy and therapeutic outcomes.

## 2. Molecular Classification

The first step to stratifying EC was conducted in the 1980s, when Bokhman described types I and II, each with distinct pathogenesis and association with hyperestrogenic status [8]. About 80% of tumors corresponded to type I and were associated with hyperestrogenism, diabetes, hyperlipidemia, and hypertension. These were low-grade carcinomas in an early stage with a more favorable prognosis. On the other hand, type II corresponded to high-grade carcinomas in the advanced stage and a more aggressive course, affecting older women without the classical epidemiological profile seen in type I [8]. The uterine serous carcinoma was described by Hendrickson et al. in 1982 [9] and became associated with the EC type II of Bokhman. The prototype of type I was endometrioid carcinoma. It did not take long for the insufficiency of this dualistic approach to become evident. In 2013, the Cancer Genome Atlas Research Network (TCGA) provided an integrated genomic, transcriptomic, and proteomic characterization of endometrial carcinomas composed of endometrioid and serous histological types. A combination of somatic mutational burden, somatic copy number alterations, and microsatellite instability allowed for the division of EC into four molecular subtypes with distinct prognosis: DNA polymerase epsilon (POLE) (ultramutated) tumors, tumors with high microsatellite instability (MSI) (hypermutated), copy-number-low tumors, and copy-number-high tumors [6]. Soon after, the Proactive Molecular Risk Classifier for Endometrial Cancer (ProMisE) was created, a tool that proposed surrogate clinically available markers for each of the four subgroups of TCGA [10]. *POLE* mutations in exons 9 to 14 correspond to POLEmut. Immunohistochemistry (IHC) testing for loss of MMR proteins identifies MSI-H, while IHC for p53 discriminates between copy-number-high (aberrant p53 expression, mutated pattern, and p53abn) and copy-number-low tumors (normal p53, p53 wild-type, and p53wt). The latter are named no specific molecular profile (NSMP) tumors [10]. Table 1 summarizes the characteristics of the four molecular subgroups of tumors.

The molecular classification of EC provides diagnostic and prognostic insights to tailor treatment decisions [15,16]. In addition to the MSI group, which corresponds to 25–30% of all tumors, which we will detail later, the identification of the POLEmut and p53abn groups contributes to strategies to support the de-escalation or escalation of treatments [15], which have even been incorporated by the 2023 FIGO staging [17].

## 3. Microsatellite Instability

Microsatellite instability (MSI) is a form of genetic hypermutability that results from impaired DNA MMR. MSI is characterized by length alterations within short repetitive DNA sequences of 1 to 10 nucleotides, denominated microsatellite or short tandem repeats, which are localized along the genome, involving coding or non-coding regions that are more prone to DNA mismatching errors. MSI-high (MSI-H) tumors, with their unique characteristics, have a high tumor mutational burden (TMB).

The TCGA study tested MSI in all samples using seven repeat loci and found this profile in 40% of carcinomas of the endometrioid type and 2% of those of the serous type [6]. These tumors presented 18 × 10^−6^ mutations/Mb, a high mutation rate at about tenfold greater than stable tumors, and were denominated the hypermutated group. The mutations seen in the samples from the MSI group differed from stable tumors; for example, they presented more *ARID5B* mutations and fewer *CTNNB1* mutations. They expressed high AKT-phosphorylated and low PTEN levels. *MLH1* promoter hypermethylation was the most common alteration and it was associated with decreased MLH1 mRNA expression [6].

## 4. The Mismatch Repair System

The MMR system is crucial for maintaining genomic stability by correcting DNA replication errors, which are more common in genome regions with short repetitive DNA sequences. The most relevant MMR proteins in humans are MLH1, MSH2, MSH6, and PMS2, which are coded by the corresponding genes. These genes are *MLH1* (mutL homolog 1), *MSH2* (mutS homolog 2), *MSH6* (mutS homolog 6), and *PMS2* (PMS1 homolog 2, mismatch repair system component), located in chromosomes 3, 2, 2, and 7, respectively, according to the Human Genome Organization (HUGO) Gene Nomenclature Committee [18]. The MMR proteins’ action mechanism involves recognizing mismatched nucleotide base pairs, excision, and repair, acting in heterodimers MLH1-PMS2 and MSH2-MSH6 [19]. The failure of this process characterizes the deficiency of MMR (dMMR), which results in MSI-H; so, the two conditions, although defined by two distinct methods, can be used interchangeably for ECs with these characteristics.

The deficiency of MMR disrupts DNA repair, leading to genetic mutations along the genome, which can affect either proto-oncogene or tumor-suppressor genes. If the dMMR is secondary to a germline MMR gene mutation, it results in an increased risk of cancer. While bi-allelic germline mutations have been described, the mono-allelic germline mutation of genes of the MMR system is the most common and corresponds to Lynch syndrome (LS) [20]. Both germline mutation and somatic alterations in the MMR system within a tumor serve as biomarkers for checkpoint inhibitors (ICIs) for recurrent/advanced disease, highlighting the potential of the MMR system in disease management.

## 5. Deficiency of Mismatch Repair/Microsatellite Instability-High (dMMR/MSI-H) as Biomarkers for Immunotherapy in Endometrial Cancer

Immunotherapy has improved the outcomes of different types of cancers. On 23 May 2017, the U.S. Food and Drug Administration (FDA) published its first tissue/site-agnostic approval for a humanized IgG4 anti-PD-1 monoclonal antibody, pembrolizumab, for unresectable or metastatic dMMR or MSI-H solid tumors that progressed following prior treatment and had no alternative treatment [21]. The decision was based on five uncontrolled multi-cohort, multicenter, single-arm, phase II clinical trial KEYNOTE-158 (NCT02628067) results. The EC cohort corresponded to 49 cases (21%), which showed an objective response rate (ORR) of 57.1% (95%CI 42.2–71.2) [22]. Let us compare the power of this result. KEYNOTE-028, a clinical trial that enrolled 24 patients selected using the PD-L1-positive status instead of dMMR/MSI-H in advanced EC, also treated with pembrolizumab, obtained an ORR of 13% (95%CI 2.8–33.6%) [23]. The update of the results of KEYNOTE-158 analyzed cohort K, corresponding to dMMR/MSI-H EC, with longer follow-up [24]. The ORR was 46% (95%CI: 35–56), and the median duration of response was not reached, with 68% of patients having response durations ≥ 12 months and 44% having response durations ≥24 months [24]. These results supported the FDA’s decision on 21 March 2022 to approve pembrolizumab for advanced EC in the second line [25]. On 9 February 2023, the FDA granted regular approval to another PD-1 inhibitor, dostarlimab, for dMMR/MSI-H advanced endometrial cancer following prior therapy [26]. The decision was based on the results of dMMR/MSI-H EC (cohort A1) from the phase I GARNET trial (NCT02715284) with a confirmed ORR of 42.3% (95%CI 30.6–54.6) [27]. In the update of GARNET with longer follow-up and 108 patients of the dMMR/MSI-H cohort, these patients were compared to those with proficient MMR and stable (MSS) (pMMR) tumors (cohort A2) [28]. The ORR was 47% (95%CI 34–53.4) and 22% (95%CI 34–53.4) in cohorts A1 and A2, respectively [28]. The response of pMMR tumors to ICIs is not negligible, which is probably related to a fraction of EC that, although stable, has other indicators of immune activations, such as PD-L1 or high TMB. For pMMR advanced EC previously treated, pembrolizumab and Lenvatinib combination therapy has excellent results, as demonstrated by the phase III study KEYNOTE-775 (randomized with the chemotherapy control group) trial [29]. Lenvatinib is a tyrosine kinase inhibitor that blocks the action of growth factors involved in cellular proliferation and angiogenesis. The ORR in patients with pMMR tumors was 32.4% in the study group and 15.1% in the controls [29].

After the enthusiastic results with PD-1 inhibitors in pre-treated advanced EC, it did not take long for these drugs to be shown to be effective in first-line treatment. Two important phase III randomized studies, RUBY (NCT03981796) [30] and KEYNOTE-868/NRG-GY018 (NCT03914612) [31], generated the data to confirm the efficacy of this indication.

The RUBY trial enrolled 494 patients for randomization: 245 to receive dostarlimab plus carboplatin–paclitaxel followed by dostarlimab, and 249 to receive placebo plus carboplatin–paclitaxel followed by placebo. The dMMR/MSI-H population had 118 patients. The benefit of dostarlimab was higher in the dMMR/MSI-H group than in the pMMR/MSS group. The 24-month progression-free survival (PFS) was 36.1% (95%CI, 29.3–42.9) in the dostarlimab group and 18.1% (95%CI, 13.0–23.9) in the placebo group (hazard ratio for progression or death, 0.64; 95%CI, 0.51–0.80; *p* < 0.001) [30].

The NRG-GY018 trial enrolled more patients, although the follow-up was smaller than RUBY’s. The entire population was 816 patients, 225 of them with dMMR/MSI-H tumors. The benefit of pembrolizumab was higher in the dMMR/MSI-H tumors. These tumors were associated with progression-free survival of 74% compared to 38% in the placebo group (hazard ratio for progression or death 0.30; 95%CI 0.19–0.48; *p* < 0.001) [31].

These two studies are very similar, although only the RUBY trial included carcinosarcomas. Again, both studies demonstrated some benefit in the pMMR group, indicating that the immune response hides other factors to be explored, which can help us better predict ICI sensitivity. This understanding contributes to stratifying the dMMR/MSI-H tumors since the response is heterogeneous in the group. In the following sections, we will discuss some factors that potentially affect the determination of dMMR/MSI-H as the ideal immunotherapy biomarker. The efficacy and safety of ICI monotherapy in EC were analyzed through a metanalysis conducted by Wan et al. [32]. This study confirmed the higher efficacy of single-agent PD-1/PD-L1 inhibitors in dMMR tumors. The ORR for patients with dMMR and pMMR tumors was 45% (95%CI, 40–50) and 8% (95%CI, 5–12). Table 2 summarizes the pivotal trials using dMMR/MSI-H as biomarkers to evaluate the ICI therapy.

## 6. Methods Used to Identify dMMR/MSI-H

Universal MMR testing is recommended for all newly diagnosed ECs. This can be performed using various techniques: immunohistochemistry (IHC) for DNA MMR proteins, polymerase chain reaction (PCR)-based assays to evaluate the length variations in microsatellite regions, or next-generation sequencing (NGS)-based MSI analyses. According to the American Society of Clinical Oncology (ASCO) and the College of American Pathologists (CAP), IHC is the recommended method for endometrial cancer [33,34]. Both Institutions agree that although dMMR/MSI-H tumors lead to TMB, this can be secondary to other conditions and should not be used as a surrogate of dMMR/MSI-H status.

**Figure 1:**
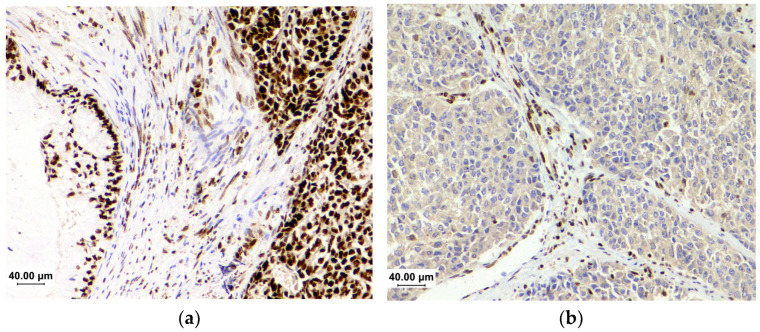
The immunohistochemistry of mismatch repair proteins in endometrial cancer. (**a**) Intact expression of MSH6—it is important to note the higher intensity in tumor cells than in stromal cells; (**b**) the loss of MLH1 expression. Tumor cells are negative, while stromal cells are positive.

IHC is based on the expression of the four DNA MMRs: MLH1, MSH2, MSH6, and PMS2. The pathologist compares the expression pattern of the tumor cells with that of adjacent cells, such as stromal and inflammatory cells, which constitute the positive internal control required to verify the reaction fidelity. Intact expression characterizes proficient tumors and is observed as homogeneous nuclear staining, which becomes more intense as the proliferative rate increases. Hence, tumor cells appear with higher staining intensity than the control cells (Figure 1a). The loss of the nuclear expression of one or more of these proteins characterizes the dMMR (Figure 1b). The tumor cells are negative in these cases, while the adjacent normal cells are stained.

MLH1 and MSH2 can stabilize within a cell by forming heterodimers with other proteins, while PMS2 and MSH6 can only stabilize with MLH1 and MSH2, respectively. Consequently, we can identify four patterns of immunostaining according to the defect: (1) MLH1 deficiency: loss of both MLH1 and PMS2; (2) MSH2 deficiency: loss of both MSH2 and MSH6; (3) MSH6 deficiency: isolated loss of MSH6; and (4) PMS2 deficiency: isolated loss of PMS2 [35]. Considering these patterns, only PMS2 and MSH6 can accurately determine the dMMR status [36]. A systematic literature review with meta-analysis demonstrated a high detection rate of dMMR (98.9%) using the two-antibody testing [37]. The authors did not find cases with isolated MLH1 or MSH2 loss or combined MLH1/MSH2 loss alone among six articles investigating the two-antibody testing. However, they recommend testing all four proteins in doubtful cases.

The concordance between IHC MMR and the MSI group of TCGA is remarkably high, instilling trust in the method. A systematic review conducted by Raffone et al. included five studies with 1110 patients for metanalysis [36]. The accuracy of IHC in detecting MSI was very high, with an area under the curve (AUC) of 0.988. The pooled sensitivity was 0.96 (95%CI, 0.93–0.98), the pooled specificity was 0.95 (95%CI, 0.93–0.96), and the pooled positive and negative likelihood ratios were, respectively, 17.7 (95%CI, 11.9–26.33) and 0.05 (95%CI, 0.01–0.2).

**Figure 2:**
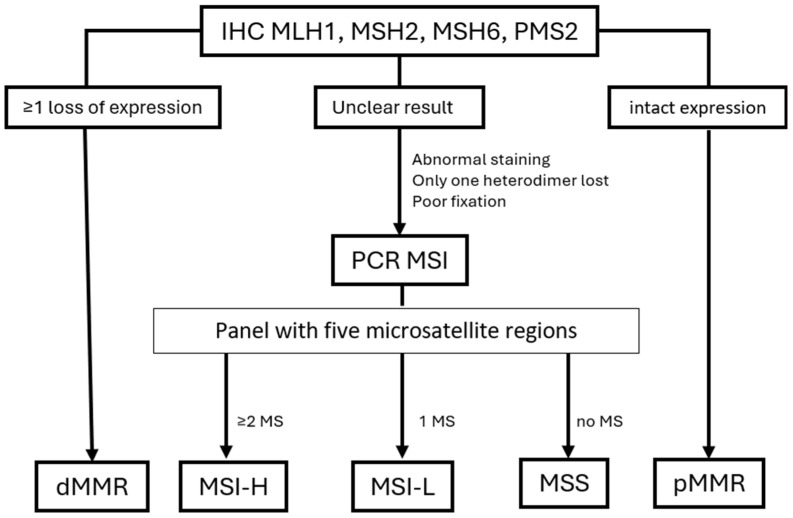
The testing flowchart used to determine the MMR/MSI status in endometrial cancer, based on the work of Noh et al. [39].

The PCR method for detecting MSI involves amplifying microsatellite regions using specific primers, separating the PCR products by gel electrophoresis, and analyzing the size variations in the microsatellite markers to determine the MSI status of the tumor. The MSI-H phenotype is identified when a particular threshold of mismatches is observed depending on the panel used. The Bethesda panel consists of two mononucleotide loci (big adenine tract BAT-25 and BAT-26) and three dinucleotide loci (D2S123, D5S346, and D17S250). The pentaplex panel (Promega™ kit) consists of five mononucleotide repeats: BAT-25, BAT-26, NR-21, NR-24, and NR-27. In both systems, tumors with instability of two or more loci are considered as MSI-H, and those without instability at any of the five loci are considered microsatellite stable (MSS). Tumors with only one unstable locus are classified as MSI-low (MSI-L) [38]. Figure 2 illustrates the testing flowchart used to determine the MMR/MSI status.

Stelloo et al. studied 854 EC cases from the PORTEC-1 and -2 clinical trials and selected 696 cases where combined IHC MMR and PCR MSI was possible [40]. They found frequencies of 74%, 24%, and 2% for MSS, MSI-H, and MSI-L, respectively. The concordance between IHC and PCR was 94% (kappa 0.854; 95%CI, 0.811–0.897; *p* < 0.001).

Although IHC and PCR are considered equivalent for the dMMR/MSI-H diagnosis, EC IHC is recommended as the first test by ASCO and CAP, and it was demonstrated to be more accurate than MSI PCR at identifying LS [33,34,41]. Discordant cases are discussed below. Tumors with evidence of poor fixation or doubtful IHC staining require a molecular test. Next-generation sequencing (NGS)-based MSI assays are not widely accessible, but, besides being highly concordant with IHC and PCR, they permit the definition of borderline cases and the analysis of other mutational signatures. Molecular tests (PCR or NGS) are recommended whenever IHC shows abnormal cytoplasmic or dot-like staining patterns and when only one heterodimer is lost [42].

### 6.1. Challenges in Immunohistochemical for MMR Assessment

Despite the feasibility of the IHC method, challenges remain in accurately assessing MMR status. Variability in testing protocols, the interpretation of results, and the need for high-quality tissue samples can impact diagnostic accuracy. Furthermore, the heterogeneity within dMMR/MSI-H endometrial cancers necessitates standardized approaches to ensure consistent and reliable results.

#### 6.1.1. Tissue Processing and Handling

The accuracy of IHC results is highly dependent on the proper processing and handling of tissue samples. Preanalytical factors such as the cold ischemia time, type of fixative, and fixation time significantly influence staining quality. General recommendations for pathology practice include a cold ischemia time as short as possible (no more than 60 min) and fixation with 10% neutral phosphate-buffered formalin for 6–48 h [43,44]. Over-fixation can lead to a loss of antigenicity, while under-fixation may result in insufficient antigen preservation [45]. Grillo et al. explored the impact of the fixation time on the IHC MMR and suggested that 24 h of formalin fixation at 4 °C is the best option [45]. Additionally, antigen-retrieval methods designed to unmask epitopes must be carefully optimized to ensure effective antibody binding.

Standardizing fixation and processing protocols is crucial to minimize variability and ensure the reliability of laboratory methods. Recent guidelines stress the need for consistent practices in tissue handling [43,45,46]. Variations in these practices among different laboratories can lead to discrepancies in test outcomes, underscoring the need for harmonized protocols to avoid such issues.

#### 6.1.2. Interpretation

Interpreting IHC results for MMR/MSI status involves assessing the presence or absence of MMR proteins in tumor tissues. However, this process is fraught with challenges related to subjective interpretation and variability in diagnostic criteria. Differences in pathologists’ experience and subjective judgment can contribute to inconsistencies in diagnosing MMR/MSI status.

False positives and negatives are significant concerns in MMR IHC testing. False positives can arise from non-specific staining, cross-reactivity with other proteins, or interpretation errors. In contrast, false negatives may result from technical issues such as poor fixation or low protein expression levels. These inaccuracies can impact clinical decision making and patient management.

Poor fixation is the leading cause of false-negative results, particularly in surgical specimens, where the cold ischemia time and delay in opening the uterus for adequate fixation are, unfortunately, still the rule in many services. To prevent this problem, MMR IHC should be conducted on the biopsy specimen [35]. Samples submitted to neoadjuvant chemotherapy or freezing are other causes of false-negative results. Correct interpretation by the pathologist, considering the pattern of staining of the normal cells, can minimize these errors.

Careful examination of tumor and stromal cells is crucial for an adequate diagnosis. Some missense mutations can result in weak/focal expression. Very weak staining/very focal expression with strong internal control should best be reported as “loss” with a comment or note. In this situation, the pathologist can suggest repeating the reaction in another sample or performing a PCR assay [35].

Other uncommon patterns of staining can occur and are responsible for false-positive results. Tumors from LS associated with a loss of MSH6 can have heterogenous expression and be misinterpreted as intact [47]. Other uncommon patterns include the punctate/dot-like nuclear expression and cytoplasmic/membranous staining. The absence of uniform nuclear staining with adequate internal control should be reported as abnormal, with a note referring to the less common pattern. In some cases of loss of expression, positive intratumoral infiltrating lymphocytes can be interpreted as the nuclei of tumor cells, leading to a false-positive result [35].

**Figure 3:**
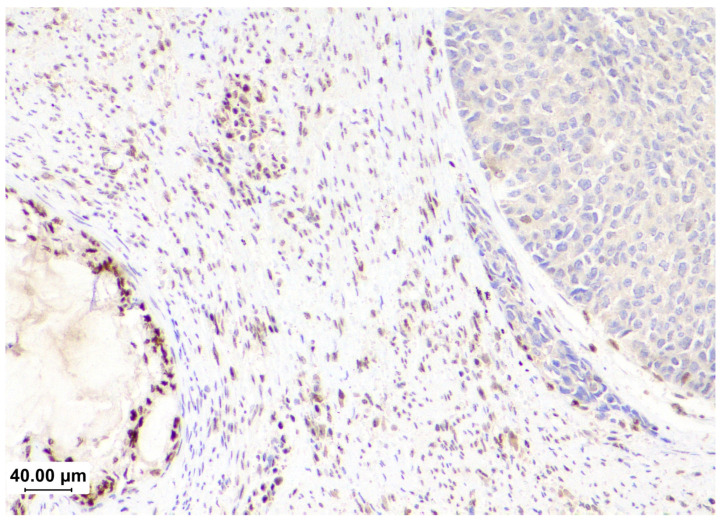
The subclonal loss of MLH1 in a case of dedifferentiated carcinoma. On the right, the undifferentiated component does not have staining, while the endometrioid component (left) and stroma show intact expression.

Subclonal loss is characterized by an abrupt and complete regional loss of staining or a reduction in staining intensity with a positive internal control [48]. This can occur in up to 8% of EC cases and is most frequently associated with MLH1 and PMS2 loss secondary to subclonal *MLH1* promoter methylation [48,49] (Figure 3).

Implementing stringent quality control measures, including appropriate controls and regular validation of staining protocols, can help mitigate false results. Additionally, complementary testing methods, such as molecular assays, can provide additional validation and support for IHC results. Table 3 shows some of these less common staining patterns and suggested approaches.

## 7. Heterogeneity of dMMR

The frequency of dMMR/MSI-H among all ECs is 30–40% of cases [6,10,11,50,51]. In our service, they corresponded to 37.2% of ECs [51]. Indeed, the group is not homogenous regarding biological behavior, including its role as a biomarker for immunotherapy. Here, we explore differences within the group.

### 7.1. Origin of the Defect

MMR deficiencies can arise from various mechanisms, including germline mutations (as seen in LS) (~10%), somatic mutations (~20%), and epigenetic silencing (e.g., MLH1 promoter hypermethylation) (~70%) [52]. These different pathways contribute to the heterogeneity observed in MSI-H endometrial cancers.

Germline mutations are associated with two syndromes: constitutional mismatch repair deficiency (CMMRD) and Lynch syndrome [42]. CMMRD is a rare and aggressive cancer predisposition syndrome in childhood due to a biallelic MMR gene mutation [53]. LS is an autosomal dominant disease caused by germline mutations in DNA MMR genes *MLH1*, *MSH2*, *MSH6*, and *PMS2*, or the *EPCAM* gene causing epigenetic silencing of *MSH2* [54].

The most common cause of dMMR/MSI-H in EC is the epigenetic silencing of MLH1 promoter hypermethylation with loss of the heterodimers MLH1 and PMS2. Immunogenicity differs according to the defect type, which interferes with the response to immunotherapy. LS-associated dMMRs have higher immune cell infiltration (CD3+, CD8+, CD45RO+, FOXP3+, and PD-L1) and higher TMB than sporadic dMMRs [55,56]. ECs with MLH1 promoter hypermethylation present lower intratumoral, stromal, and peritumoral infiltrating lymphocytes (TILs) and a lower TMB [57].

The type of protein defect can suggest the origin of the loss. Tumors with MLH1/PMS2 loss have a higher probability of an epigenetic origin, while those with MSH2/MSH6 have a higher probability of a mutational defect. In a study by Khushman et al., EC with MSH2/MSH6 loss presented a higher neoantigen load and better prognosis than EC that is MLH1/PMS2-deficient [58]. TMB, an important indicator of immune activation, was evaluated in 1057 MSI-H tumors of different histological types and was higher in the MSH2/MSH6 loss subgroup than in MLH1/PMS2 [59].

The clinicopathological characteristics and prognosis also differ according to the origin of the defect. In the study by Cosgrove et al., MMR defect associated with *MLH1* hypermethylation presented with a more advanced stage, higher grade, more lymphovascular space invasion, and older age [60]. Similar results were observed by Manning-Geist et al. in their study of 181 patients with MSI-H EC from a cohort of >1100 EC patients [57]. Kaneko et al. compared the clinical features of tumors with MLH1 promoter hypermethylation to those with losses in MMR that are non-methylated [61]. They demonstrated not only more aggressive tumors (higher grade and stage) but poorer prognosis among patients with the hypermethylated MLH1 promoter. In our previous study, although we did not perform molecular testing to prove hypermethylation of the MLH1 promoter, we observed that MLH1/PMS2 loss compared with MSH2/MSH6 loss presented a more advanced stage (44% vs. 19.7%, *p* = 0.005), larger tumors, deeper myometrial invasion, more lymphovascular space invasion, and more positive lymph nodes [51]. In our cohort, dMMR tumors had a better prognosis than pMMR. Although MLH1/PMS2 loss tumors were associated with more aggressive features, they did not have a poorer prognosis than MSH2/MSH6, suggesting that other factors can be involved in a favorable prognosis, possibly immune activation secondary to the mismatch defect. In this same line, Ma et al. proposed a subclassification of dMMR EC according to deficiency: MutL (MLH1/PMS2) and MutS (MSH2/MSH6). MutS tumors were associated with better prognosis and immune status and better immunotherapy response [62].

Table 4 summarizes the main differences between the three groups of dMMR/MSI-H according to the origin of the defect.

### 7.2. Mismatch Repair/Microsatellite Instability Discordance

Discordances between IHC and PCR are uncommon, as reported in 5–10% of endometrial cancers [63]. Riedinger et al. presented data from a cohort of 666 cases and found a discordant rate of 3.8%. They observed that most cases (72%) had less common staining patterns, mainly subclonal loss or heterogeneity [47]. In this subgroup, epigenetic loss was observed in 66.7%, germline mutations in 16.7%, and somatic mutations in 16.7%. The recurrence rate was higher among subclonal/heterogenous dMMR cases, and their metastases were dMMR. This finding indicates that tumors with subclonal loss behave as dMMR/MSI-H, stressing the need to test the metastasis of tumors with less common staining patterns. Ta et al. described 29 patients with paired primary and metastatic/recurrent EC. They found two (6.9%) cases of discordance, both with subclonal loss [64]. Smithgall et al. analyzed the two areas of a tumor with subclonal loss separately and observed that the area with dMMR was MSI-H and that MMRp was stable [63]. These factors demonstrate the importance of choosing adequate samples to analyze and re-test the metastatic/recurrent tumor.

### 7.3. Association with POLE Mutation and/or p53-Mutated

Some ECs present more than one molecular feature (dMMR/MSI-H, POLEmut, or p53-mutated [65,66]. Multiple classifiers include tumors POLEmut-dMMR, POLEmut-p53abn, dMMR-p53abn, and POLEmut-dMMRd-p53abn. Their frequency in the literature varies from 1.8% to 14.3%, according to a review conducted by De Vitis et al. [66]. Initially, the analysis of somatic nucleotide and copy number variations in TCGA, coupled with the outcome, supported the categorization of tumors harboring POLE mutations and dMMR combined with p53abn as the respective single classifier (POLEmut or dMMR) [65]. However, more recently, with the inclusion of *TP53* mutations in addition to p53 IHC resulting in a higher frequency of this combination, it seems that dMMR-p53abn ECs have characteristics intermediate between dMMR and p53abn subtypes [66]. A study by Kato et al. included 337 Japanese patients with EC tested with IHC for MMR. Protein loss was seen in 91 (27%) patients, 13 with LS. The overall survival rate among patients with LS was 100%, without significant differences compared to the group with sporadic dMMR. However, the sporadic dMMR and p53abn subgroup presented a 5-year overall survival significantly worse than that in patients with p53wt (53.6% vs. 93.9%, log-rank test *p* = 0.0016) [67]. These results suggest that the association with p53abn, even if these tumors have defects in MMR proteins and are classified as dMMR, may impact the response to immunotherapy.

The dMMR-POLEmut association is much less frequent and much more complex. León-Castillo et al., in a pooled analysis that included 3361 ECs, found 13 cases with dMMR associated with a pathogenic *POLE* exonuclease domain mutation (EDM) and 14 with non-pathogenic POLE EDM. The five-year recurrence-free survival rates were 92.3% and 76.2%, respectively, indicating that the interpretation of POLE mutations is the guide for prognosticating EC. The POLEmut definition can be determined by identifying any of the 11 POLE EDM described in TCGA or by a POLE score developed by the authors that predicts the ultramutated state [68].

In the GARNET trial (NCT2715284), to evaluate the antitumor activity of the anti-PD1 dostarlimab, five cases of POLEmut were used, two of them with strong responses, but both were also dMMR/MSI-H [69]. This suggests that the immunotherapy response is related to the dMMR status and is not influenced by the POLE status.

### 7.4. Association with Other Immune Biomarkers

The consequence of the repair defects present in dMMR/MSI-H EC is the generation of neoantigens, which lead to immune activation. This activation can be appreciated in tumors through several biomarkers, such as TMB, tumor-infiltrating lymphocytes (TILs), and PD-L1.

TILs are more numerous in dMMR/MSI-H and POLEmut than in p53 and in the no specific molecular profile (NSMP) [15]. As expected with the high population of TILs, the expression of PD-L1 in dMMR EC is also high. Favier et al., in their systematic review of biomarkers in endometrial tumors with dMMR, found four articles demonstrating PD-L1 higher than in pMMR [70]. However, the high TIL population is not the rule for all dMMR/MSI-H EC. Those defects originated by mutations are associated with a higher TIL population and better responses to immunotherapy [56,71].

The phase I GARNET trial (NCT2715284), which aimed to evaluate the antitumor activity of the anti-PD1 dostarlimab, explored therapeutic responses according to TMB and PD-L1 expression [69]. High TMB (TMB-H) was more frequent among dMMR/MSI-H tumors (86.5% vs. 7.2%); however, when present, it was associated with efficacy in both subgroups. The ORR among TMB-H tumors was 47.5%; 95%CI 37.5–57.7), independent of the MMR status. The cohort of dMMR/MSI-H tumors presented an ORR of 54.9% (95%CI 43.5–65.9) in the PD-L1-positive (CPS ≥ 1) subgroup and 31.3% (95%CI 16.1–50.0) in the PD-L1-negative subgroup (CPS < 1). The combination of TMB with CPS showed exciting results. TMB-H plus PD-L1-positive tumors had the highest ORR, independent of the MMR/MSI status, higher than 60%. When both biomarkers were negative, the ORR was low in both cohorts, but mainly in pMMR/MSS tumors (7.1% vs. 20%), suggesting that the MMR status per se can improve the therapeutic response, albeit to a lesser extent. These results indicate that a single biomarker is insufficient for therapeutic decisions.

In another clinical trial (NCT03241745), a phase II study using another PD-1 inhibitor, nivolumab, the authors explored the immune cells of the microenvironment in dMMR/MSI-H advanced or recurrent endometrial and ovarian cancer [72]. This trial included all uterine tumors, including sarcomas and ovarian cancer of a histology common to endometrial tissue, such as carcinosarcoma, endometrioid carcinoma, and clear-cell carcinoma. The exploratory analysis of biomarkers suggests that the functional profile of T cells and the spatial distribution of CD8+ and PD-L1+ cells better stratify dMMR/MSI-H tumor candidates for anti-PD-1 therapies.

## 8. Conclusions

**Figure 4:**
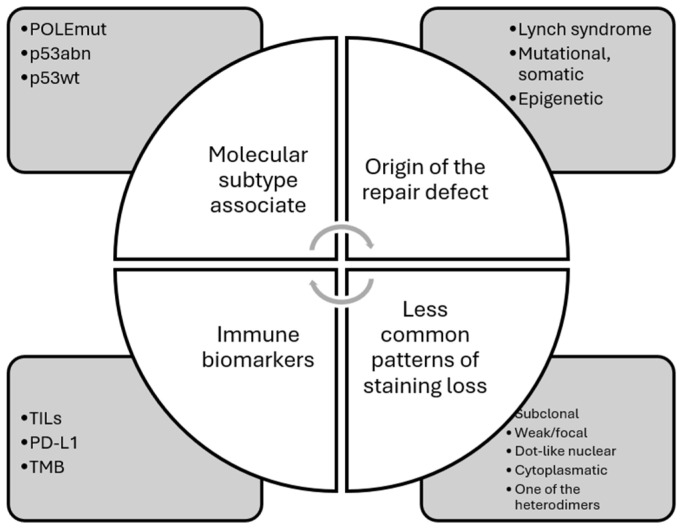
The many facets of deficient mismatch repair/microsatellite instability-high endometrial carcinomas.

Understanding the heterogeneity of mismatch repair protein deficiencies in endometrial cancer is essential for accurate diagnosis and personalized treatment. In Figure 4, we illustrate the multiple facets of the dMMR/MSI-H in EC. Advancements in diagnostic techniques and more profound knowledge of the molecular underpinnings of these cancers will enhance our ability to tailor therapies and improve outcomes for patients with MSI-H/dMMR endometrial cancer.

## 9. Future Directions

The effectiveness of immunotherapy based on PD-1/PD-L1 blockage as single agents is sufficiently demonstrated, but patient responses are not homogeneous. The next steps include subclassifying dMMR/MSI-H tumors beyond simply “loss” or “intact” and investigating other predictive biomarkers to benefit from adding new drug options according to these subtypes.

## Figures and Tables

**Table 1:** The molecular subtypes of endometrial carcinomas according to the Cancer Genome Atlas Research Network (TCGA) and their main clinicopathological features.

TCGA Subgroups [6]	POLE	MSI ^1^	CN-H ^2^	CN-L ^3^
ProMisE ^4^ surrogates [10]	Exons 9–14 mutations	dMMR ^5^	p53-mutated	p53-wild-type
Frequency [6]	7%	28%	26%	39%
Age at diagnosis < 60 y [11]	57.1%	38.3%	6.6%	51.4%
BMI [12] ^6^	27.2 ± 0.9	30.6 ± 1.2	29.1 ± 0.5	32.3 ± 1.4
High-risk ESMO (2016) ^7^ [10]	16.7%	33.9%	87.3%	14.5%
FIGO ^8^ stage I (2009) [10]	92.9%	78%	52.7%	86.8%
Positive lymph node [11]	14.2%	14.9%	44.8%	10.8%
Endometrioid histology [13]	86.1%	85.8%	27%	96.7%
High-grade tumor (grade 3) [11]	23.8%	12.8%	93.3%	6.8%
TILs ^9^ [14]	high	high	absent	low
LVSI ^10^ [11]	28.6%	34%	20.3%	60%
*TP53* mutation [6]	35%	5%	>90%	1%
Prognosis [6,10]	excellent	intermediate	poor	intermediate

^1^ MSI: microsatellite instability; ^2^ CN-H: copy number-high; ^3^ CN-L: copy number-low; ^4^ ProMisE: Proactive Molecular Risk Classifier for Endometrial Cancer; ^5^ dMMR: deficient mismatch repair; ^6^ BMI: body mass index; ^7^ ESMO: European Society of Medical Oncology; ^8^ FIGO: Internation Federation of Gynecology and Obstetrics; ^9^ TILs: tumor-infiltrating lymphocytes; ^10^ LVSI: lymphovascular space invasion.

**Table 2:** The pivotal studies that explored the efficacy of immune checkpoint inhibitors in advanced or recurrent deficient mismatch repair/microsatellite instability-high (dMMR/MSI-H) endometrial cancer.

Clinical Trial	Type of Study	n	Treatment	Main Result
KN-158 NCT02628067 [22]	Single-arm, phase II study	49	Pembrolizumab	ORR 57.1% (95%CI 42.2–71.2)
GARNET NCT02715284 [27]	Phase I, single-arm	104	Dostarlimab	ORR 42.3% (95%CI 30.6–54.6%)
KN-868/NRG-GY018 NCT03914612 [31]	Phase 3, randomized, placebo control	222	Pembrolizumab + carboplatin/paclitaxel followed by pembrolizumab	PFS 74% vs. 38%
RUBY NCT03981796 [30]	Phase 3, randomized, placebo control	118	Dostarlimab + carboplatin/paclitaxel followed by dostarlimab	PFS: 61.4% vs. 15.7% OS: 36.1% vs. 18.1%

ORR: objective response rate; PFS: progression-free survival; OS: overall survival.

**Table 3:** Less common staining patterns exhibited in immunohistochemistry for mismatch repair proteins, their probable meaning, and suggestions for actions that can be performed by pathologists in the report (based on the work of Singh et al. [35]).

Pattern of Staining	Interpretation	Action
Subclonal loss with adequate internal control in both areas	Loss	Comment and/or PCR for MSI
Negative staining in both TC and internal control	Inconclusive	PCR for MSI or repeat test in other samples
Weak/focal staining in TC and internal control	Inconclusive	PCR for MSI or repeat test in other samples
Weak/focal staining in TC and strong staining in internal control	Loss	Comment
Punctate/dot-like nuclear staining with adequate internal control	Loss	Comment and/or repeat test in other samples or PCR for MSI
Cytoplasmic staining with adequate internal control	Loss	Comment and/or repeat in other samples or PCR for MSI

**Table 4:** A comparison of dMMR/MSI-H according to the origin of the mismatch repair defect.

Features	Mutation	No Mutation
Type of defective protein	MSH2/MSH6	MLH1/PMS2
Age of patient	younger	older
Tumor size	smaller	larger
Tumor grade	low	high
LVSI *	less	more
Stage	early	advanced
TILs, PD-L1, TMB	high	less

* LVSI: lymphovascular space invasion.

## Data Availability

No new data were created or analyzed in this study. Data sharing is not applicable to this article.
